# Nitric Oxide Releasing Polymeric Coatings for the Prevention of Biofilm Formation

**DOI:** 10.3390/polym9110601

**Published:** 2017-11-11

**Authors:** George Fleming, Jenny Aveyard, Joanne L. Fothergill, Fiona McBride, Rasmita Raval, Raechelle A. D’Sa

**Affiliations:** 1 Department of Mechanical, Materials and Aerospace Engineering, University of Liverpool, Liverpool L69 3GH, UK; sggflemi@liverpool.ac.uk (G.F.); zippy78@liverpool.ac.uk (J.A.); 2 Institute of Infection and Global Health, University of Liverpool, 8 West Derby Street, Liverpool L69 7B3, UK; jofoth@liverpool.ac.uk; 3 The Open Innovation Hub for Antimicrobial Surfaces, Surface Science Research Centre, Department of Chemistry, University of Liverpool, Liverpool L69 3BX, UK; fmcbride@liverpool.ac.uk (F.M.); raval@liverpool.ac.uk (R.R.)

**Keywords:** nitric oxide donors, *N*-diazeniumdiolates, drug release, antimicrobial surfaces, biofilm prevention

## Abstract

The ability of nitric oxide (NO)-releasing polymer coatings to prevent biofilm formation is described. NO-releasing coatings on (poly(ethylene terephthalate) (PET) and silicone elastomer (SE)) were fabricated using aminosilane precursors. Pristine PET and SE were oxygen plasma treated, followed by immobilisation of two aminosilane molecules: *N*-(3-(trimethoxysilyl)propyl)diethylenetriamine (DET3) and *N*-(3-trimethoxysilyl)propyl)aniline (PTMSPA). *N*-diazeniumdiolate nitric oxide donors were formed at the secondary amine sites on the aminosilane molecules producing NO-releasing polymeric coatings. The NO payload and release were controlled by the aminosilane precursor, as DET3 has two secondary amine sites and PTMSPA only one. The antibacterial efficacy of these coatings was tested using a clinical isolate of *Pseudomonas aeruginosa* (PA14). All NO-releasing coatings in this study were shown to significantly reduce *P. aeruginosa* adhesion over 24 h with the efficacy being a function of the aminosilane modification and the underlying substrate. These NO-releasing polymers demonstrate the potential and utility of this facile coating technique for preventing biofilms for indwelling medical devices.

## 1. Introduction

Bacterial adhesion followed by biofilm formation at an implantation site can pose a significant health risk for patients with indwelling medical devices. The longevity and viability of these tissue-contacting devices are highly reliant on modifying the material surface properties to impart antimicrobial function. Within the National Health Service (NHS), approximately 300,000 patients acquire healthcare-associated infections (HCAIs) annually, with susceptibility to these increasing when devices are implanted [1]. These HCAIs are of significant economic burden to health services and are linked with increased patient morbidity and mortality [2,3]. The most frequent HCAIs detected were respiratory tract (22.8%), catheter associated-urinary tract (17.2%) and surgical site infections (15.7%) [4]. Owing to the prevalence of these device-related infections, there has been much focus on developing antimicrobial coatings that can eliminate bacterial adhesion and subsequent biofilm formation at the implantation site.

When bacteria first interact with a surface, they are in a planktonic state, which results in a rapid, non-specific, reversible colonization of the surface [5]. If bacteria attach irreversibly, a phenotypic change is triggered as a biofilm is formed. The pattern of gene expression for a planktonic bacterial cell adhering to the surface is significantly different (up to 70%) to one in a biofilm phenotype. Biofilm bacteria irreversibly anchor to the surface and possess mechanisms that allow them to evade immune responses of the host, thus increasing their virulence [6,7]. This increase in virulence and resistance to antimicrobial therapies makes treating HCAIs challenging. *Pseudomonas aeruginosa* is a Gram-negative bacterial pathogen frequently found in water, soil and plants [8,9] that can cause serious infections in hosts such as humans, plants and animals [8,10]. It has been classed by the World Health Organization (WHO) as one of the top three priority pathogens worldwide in urgent need of new antibiotics for treatment [11]. It is the most commonly-isolated organism from patients with hospital stays of one week or more, as well as one of the leading causes of nosocomial infections, worldwide [12,13]. 

The most commonly-used strategy in terms of infection control for biomaterials and medical devices is to incorporate antimicrobials into coatings in order to kill planktonic cells before a biofilm can be formed at the implant site [14]. Importantly, these coatings must also balance the antimicrobial efficacy against the growing epidemic of antimicrobial resistance. A promising strategy for the next generation of antimicrobial coatings will be to specifically target the bacteria’s signalling pathways affecting biofilm formation and detachment. Theoretically, by disrupting bacterial signalling pathways, there should be a lower tendency for the bacteria to develop defence responses and resistant mutants. The use of a “universal” antibiofilm molecule for biofilm prevention and dispersal would be ideal, but this remains elusive. The most promising strategies would borrow from nature as they have evolved over millions of years and are still effective [15,16].

NO is a diatomic free radical produced endogenously and has a crucial role in wound healing and neurotransmission and is an innate host antimicrobial response against viruses, bacteria, parasites and fungi [17,18,19,20]. The mechanism of action of NO results from its interaction with superoxide and oxygen to form reactive nitrogen species that exhibit bactericidal properties through DNA cleavage, lipid peroxidation and protein dysfunction [19,21]. As a result of NO having several mechanisms of bacterial inactivation, it is regarded as a broad-spectrum antimicrobial with a low tendency towards developing resistance mechanisms [19,21]. Moreover, very low levels of NO (nM) have been shown to prevent biofilm formation and dispersal via a signalling pathway [22,23]. 

**Figure 1:**
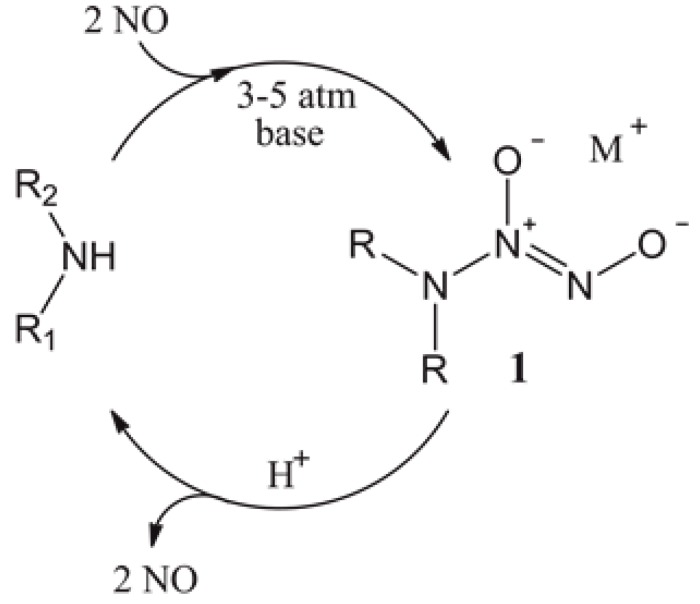
Synthesis and decomposition of diazeniumdiolates.

To be considered for use in the clinical setting, NO donors must be capable of controlled NO release at the required site and be stable for storage [24]. Despite the fact that NO is a potent therapeutic and antimicrobial agent, devising a means of storing NO is technically challenging due to its high reactivity and short half-life [24,25]. Several types of NO donors that are capable of releasing NO under physiological conditions have been reported: *N*-diazeniumdiolates, *S*-nitrosothiols (RSNOs), organic nitrates and nitrites and NO-metal complexes [19,26,27,28,29,30,31,32]. Out of these, *N*-diazeniumdiolates and RSNOs are the most widely-employed NO donors as they spontaneously decompose under physiological conditions. When exposed to proton sources, such as water or buffer, the *N*-diazeniumdiolates decompose to regenerate NO as seen in Figure 1 [32]. 

First identified by Drago, *N*-diazeniumdiolates (**1**) are compounds containing the functional group [N(O)NO], formed as a product of exposing secondary amines to high pressures of NO [33,34]. *N*-diazeniumdiolates have shown great potential in a variety of medical applications, requiring the rapid or gradual production of NO as they are stable as solid salts, but theoretically release two moles of NO when dissolved in aqueous solution at physiologically-relevant conditions, as seen in Figure 1 [35,36]. By varying the amine precursor, *N*-diazeniumdiolates can be synthesized with tuneable NO release rates with half-lives ranging from 2 s–20 h [37]. The addition of NO donors into polymeric materials has been demonstrated to be non-cytotoxic and non-haemolytic and preserves the mechanical properties of the underlying substrate polymer [38]; however, there is still a need to control the payload and external long-term release of the coating, whilst also preventing leaching. Meyerhoff’s group has demonstrated that doping lipophilic dibutyhexyldiamine diazeniumdiolate into polymer films can be used as stable NO donors with minimal leaching [39]. The advantage of such a system is that the proton-catalysed release mechanism of NO creates free lipophilic amine species, which increases the pH, consequently slowing the NO release. Schoenfisch studied a range of NO-releasing xerogel and sol-gel polymers that are capable of inhibiting bacterial adhesion [40,41,42]. Unfortunately, the xerogels have had problems with stability due to the exposure of high pressures of NO during *N*-diazeniumdiolate formation, which appears to enhance sol-gel polycondensation, producing dense and non-permeable xerogel coatings [43]. Schoenfisch’s group has also developed NO-releasing dendritic scaffolds using *N*-diazeniumdiolate derived from primary amine, secondary amine and amide functionalities. The secondary amine had the highest payload/storage capacity for NO owing to higher stability of the secondary amine diazeniumdiolates [44]. These macromolecular dendritic NO scaffolds had half-lives that significantly surpassed those for small molecule equivalents [44]

Herein, we report on the development and antimicrobial efficacy of stable NO-releasing polymer coatings on two medically-relevant polymers used for indwelling medical devices (poly(ethylene terephthalate) (PET) and silicone elastomer (SE)) and their efficacy in preventing *Pseudomonas aeruginosa* biofilm formation. The NO-releasing polymer coatings are synthesised as covalently tethered aminosilane-precursor diazeniumdiolates in order to yield a range of NO-release properties. The purpose of the study was to evaluate the relationship between the structure of the precursor aminosilane used and its relationship to decomposition rates and biofilm prevention. Much of the research looking at efficacy of antimicrobial coatings studies microbes in planktonic, nutrient-rich batch cultures, which is good for initial in vitro screening; however, in vivo infections are typically caused by bacterial biofilms [45,46]. In this study, we look at the antimicrobial efficacy by using overnight cultures of *P. aeruginosa* allowed to grow for 24 h at pH 7.4 under static conditions. Clinical isolate *P. aeruginosa* (PA14) was selected for biofilm prevention studies, as they are well-characterized medically-relevant opportunistic bacteria that form biofilms [47].

## 2. Materials and Methods

### 2.1. Preparation of Polymer Substrates for Analysis

Sheets of poly(ethylene terephthalate) (PET), 0.175 mm thick, and silicone elastomer (SE), 1 mm thick (Goodfellow, Cambridge, U.K.), were used as the substrate materials. Disks of each polymer, 6 mm in diameter, were used in NO release quantification experiments and bacterial assays. For all other analyses, substrates were cut into 15 × 15 mm squares. Pristine substrates were subjected to oxygen (BOC, Guildford, U.K.) plasma treatment at a gas flow rate of 14 standard cubic centimetres per minute (sccm) and a pressure of 0.75 mbar using a HPF100 plasma treatment system (Henniker Plasma, Warrington, U.K.).

### 2.2. Preparation of Aminosilanised Substrates

**Figure 2:**
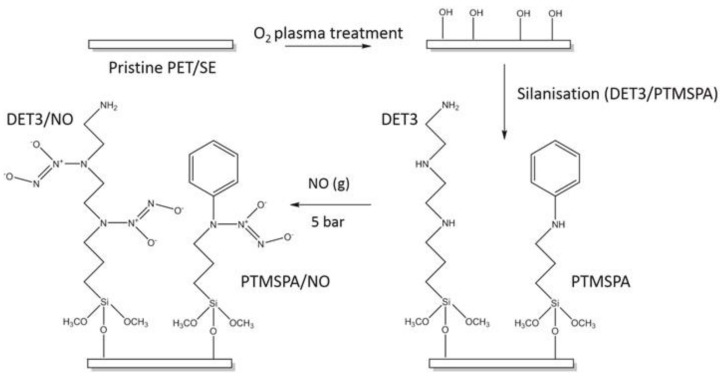
Reaction scheme for tethering of diazeniumdiolates onto PET and silicone elastomer (SE). DET3, *N*-[3-(trimethoxysilyl)propyl]diethylenetriamine; PTMSPA, *N*-[3-trimethoxysilyl)propyl]aniline.

Substrates were aminosilanised using *N*-(3-(trimethoxysilyl)propyl)diethylenetriamine (DET3) and *N*-(3-(trimethoxysilyl)propyl)aniline (PTMSPA) (Sigma-Aldrich, St. Louis, MO, USA), (Figure 2). Briefly, pristine PET and SE were subjected to oxygen (BOC, Guildford, U.K.) plasma for optimum treatment times of 7 and 2 min, respectively, as determined from preliminary experiments. Immediately after treatment, substrates were immersed in 10% solutions of either DET3 or PTMSPA in EtOH for 2 h. Substrates were rinsed in anhydrous EtOH, dried in air and cured for 4 h at 80 °C.

### 2.3. Preparation of Diazeniumdiolate-Tethered Substrates

Diazeniumdiolate tethering was carried out using a stainless steel reactor built in-house. Silanised substrates were placed into the reactor and the system purged with 6 bar of argon (BOC, Guildford, UK) for 3 × 5 min and 3 × 10 min. The reactor was then filled with 5 bar of nitric oxide (NO) (BOC, Guildford, UK) for 96 h. Upon release of NO from the system, the system was purged with 6 bar of argon for 2 × 5 min and 2 × 10 min. Substrates were removed from the reactor and stored at −20 °C prior to use.

### 2.4. Contact Angle Analysis

Static contact angles of water were used to determine changes in surface wettability following each step of the synthesis, using an Attension ThetaLite optical tensiometer (Biolin Scientific, Västra Frölunda, Sweden). The sessile drop method was used, and contact angles were taken at 17 frames per second for 10 s and data recorded using OneAttension software (Biolin Scientific, Västra Frölunda, Sweden). At least three readings were performed per sample type and the results recorded as the mean average ± the standard deviation.

### 2.5. XPS Analysis

XPS analysis was carried out on an Axis-Supra instrument (Kratos Analytical, Manchester, UK) using a monochromated Al Kα X-ray source operating at a power of 225 W. Charge compensation was performed using a low-energy electron flood source. Survey and narrow region scans were carried out at pass energies of 160 and 20 eV and step sizes of 1 and 0.1 eV, respectively. Hybrid lens mode was used in both cases. Data were converted to vamas (*.vms) format and analysed using CasaXPS 2.3 software (Casa Software, Devon, UK). Spectra were calibrated to 284.6 and 285.0 eV for SE and PET, respectively, corrected with linear background removal and fitted using Gaussian-Lorentzian line curves.

### 2.6. Atomic Force Microscopy

AFM was used to observe changes in surface topography occurring during synthesis. A Bruker Multimode 8 (Bruker, Billerica, MA, USA) system fitted with a NanoScope V controller was used, and samples were imaged in air in ScanAsyst mode using a silicon RTESPA-150A tip operating at a scan rate of 0.9 Hz. Third order flattening was used to correct any errors whilst processing the image. 5 × 5 µm^2^ images were taken and root mean square roughness (*R*_q_) and average roughness (*R*_a_) measured using NanoScope Analysis 1.7 software.

### 2.7. Electrochemical NO Detection

NO detection was carried out using an ISO-NOPF200 NO-specific electrochemical sensor (WPI, ‎Hitchin, UK). The NO probe was maintained as per the instruction manual by routinely equilibrating it in distilled water for the purpose of acquiring stable background current measurements before samples were tested. *S*-nitroso-*N*-acetylpenicillamine (SNAP) was used to calibrate the sensor and has been recommended by WPI to be suitable for calibrating the system for long- and short-term donors [48,49]. Briefly, the sensor was left to polarise in 20 mL of 0.1 M CuCl_2_ solution. Following polarisation, calibration was carried out on a daily basis owing to the fact that these types of electrochemical sensors measure small changes in voltage and are therefore extremely sensitive to temperature fluctuations and external noise [50,51]. Upon achieving a steady baseline, aliquots of 10 µM SNAP solution (20, 40, 80, 160 and 320 µL) were added sequentially to the CuCl_2_ solution to give final concentrations of 6, 12, 24, 48 and 96 nM of NO in the solution as outlined by WPI [48,49]. Each aliquot of SNAP addition increased the voltage rapidly, followed by a plateau, which decayed before the next volume was added. SNAP releases NO with a 60% efficiency, and this conversion yield is used to create a calibration curve of voltage vs. NO concentration. For substrate measurements, the NO probe was placed in either 2 mL acetate buffer (pH 4) or PBS (pH 7), until the baseline was stable (5–10 min). Substrate disks were then placed into the solution, and measurements were taken for 30 min (pH 4) and 24 h (pH 7). All experiments were conducted in a temperature controlled room at 25 °C. In order to confirm that the electrochemical sensor was measuring NO release, 100 μM of the NO scavenger 2-4-carboxyphenyl-4,4,5,5-tetramethylimidazoline-1-oxyl-3-oxide (cPTIO, Sigma , Dorset, UK) was added after the NO release measurements. The addition of this NO scavenger resulted in a decrease of the response back to the baseline establishing that only NO was measured. 

### 2.8. Biofilm CFU Assay

Antimicrobial tests were carried out against the *P. aeruginosa* laboratory reference strain PA14 [52]. Overnight cultures of *P. aeruginosa* were diluted to McFarland Standard 0.5 in Luria-Bertani (LB) broth. Substrate disks were placed in 24-well plates and 2 mL of the bacterial solution added before incubating at 37 °C for 24 h to allow biofilm formation. At the end of this time, substrate disks were transferred to sterile well plates and washed with PBS to remove any non-adhered planktonic bacteria. Substrates were then placed in fresh wells and repeatedly washed and agitated vigorously to remove and re-suspend the attached biofilm. A serial dilution was performed on LB agar using the Miles and Misra method in order to enumerate the bacteria from the biofilm. All samples were studied in triplicate and repeated five times.

### 2.9. Statistical Analysis

The statistical analysis of bacterial numbers was performed using the data analysis package, SigmaPlot 13.0 (Systat Software, San Jose, CA, USA). One-way analysis of variance (ANOVA) was used to establish differences between group means and thus variance between treatment types. Significance between treatment types was determined using the Student–Newman–Keuls (SNK) method. A value of *p* < 0.05 was taken as statistically significant.

## 3. Results

The synthesis of diazeniumdiolates onto PET and SE is outlined in Figure 2. Briefly, pristine PET and SE were plasma treated to introduce oxygen functionalisation onto the surfaces. This was followed by silanization with the monoamine (PTMSPA) and the triamine (DET3). The aminosilane surfaces were then exposed to a high pressure of NO for 96 h to form the *N*-diazeniumdiolate. 

### 3.1. Surface Wettability: Contact Angle

The average contact angle values after each synthesis step are recorded in Table 1. A steep reduction in contact angle was observed on both substrates after plasma treatment, when compared with the corresponding pristine controls, confirming an increase in wettability as a result of the oxygen functionalisation of the surfaces. An increase in contact angle was also observed on all substrates following silanisation of PET-DET3 (90.2°), PET-PTMSPA (88.5°), SE-DET3 (116.6°) and SE-PTMSPA (119.2°) (Table 1), which confirmed functionalisation. After tethering the diazeniumdiolate, there was a slight decrease from the aminosilane control surfaces for both PET (PET-DET3/NO (79.0°), PET-PTMSPA (81.6°)) and SE surfaces (SE-DET3/NO (108.8°), SE-PTMSPA (108.6°)). This minor increase in wettability is most likely due to the diazeniumdiolates being more polar, as well as decomposition products (NO_2_^−^ and NO_3_^−^) caused by the water droplet of the contact angle measurement. 

### 3.2. XPS Analysis

#### 3.2.1. PET

**Figure 3:**
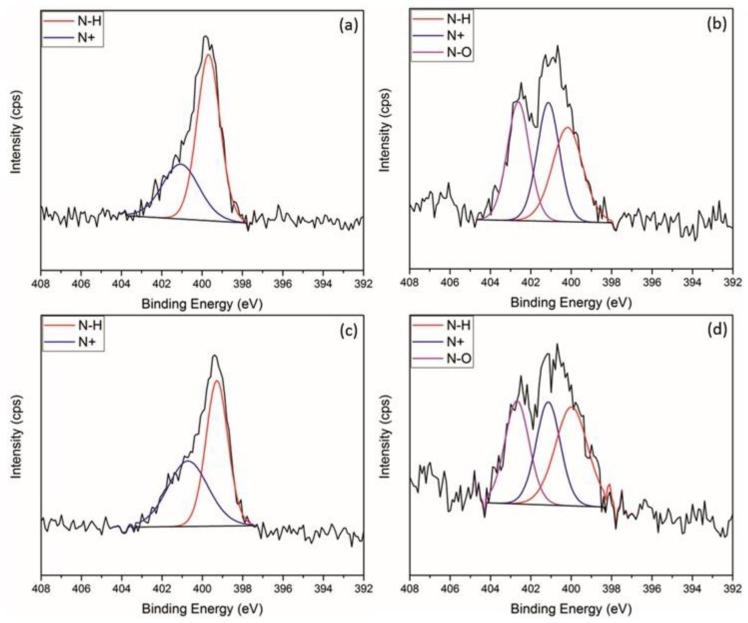
Curve-fitted N 1s XPS spectra for (**a**) PET-DET3, (**b**) PET-DET3/NO, (**c**) SE-DET3 and (**d**) SE-DET3/NO.

The success of each synthetic step for PET as a substrate was followed by XPS, and the resulting quantitative data are given in Table 2 and Table 3. Curve fitting of the C 1s envelope of PET samples gave three components: C–C/C–H at 285.0 eV, O–CH_2_CH_2_ at 286.5 eV and O=C–O at 288.9 eV. PETox C 1s spectra were fitted in the same way. After plasma treatment, an increase in the O 1s peak was observed from 27–34 at %. This is attributed to an increase in oxygen functionalisation as evidenced from the curve fitting of the C1s envelope, which showed a decrease in the aromatic/aliphatic components commensurate with an increase in ether and ester type functional groups as seen in Table 3 (increase of 13.4% for O–CH_2_CH_2_ and 15.0% for O=C–O). Immobilisation of the aminosilanes onto PETox was confirmed by the appearance of the N 1s and Si 2p peak. Tethering of the diazeniumdiolate on the surface did not change the overall elemental compositions. However, curve fitting the high resolution N1s spectra clearly shows an additional component at 402.5 eV indicative of an N-O bond from the diazeniumdiolate moiety (Table 3, Figure 3a,b).

#### 3.2.2. SE

The success of each synthetic step for SE as a substrate was followed by XPS, and the resulting quantitative data are given in Table 2 and Table 4. The C 1s envelope of SE was curve fitted to give one component at binding energy 284.6 eV characteristic of C–H/C–C/C–Si. The high resolution Si 2p peak was curve fitted at 102.1 and 103.0 eV, indicative of R_2_-Si(O)_2_ and R-Si(O)_3_.Upon plasma treatment, the at % of oxygen goes up slightly, from 32.3%–35.1%. Peak fitting of the C 1s envelope shows two components, one at 284.6 and one at 285.7 eV (C–O). The Si 2p spectra now have an extra component at 104.0 eV characteristic of Si(O)_4_ groups. SE is known to undergo rapid hydrophobic recovery after plasma treatment [53]. The most widely-accepted mechanism for hydrophobic recovery is the formation of an inorganic silica layer, which is covered by low molecular weight species that have diffused from the polymer bulk, which is consistent with what is observed here [53]. Similarly to PET, immobilisation of the two aminosilane molecules is observed by the introduction of the N 1s peak. Tethering of the diazeniumdiolate is confirmed by the appearance of a new peak in the N 1s spectrum at 403.1 eV, which is indicative of the N–O bond in the (Table 4, Figure 3c,d). 

### 3.3. Atomic Force Microscopy

#### 3.3.1. PET

**Figure 4:**
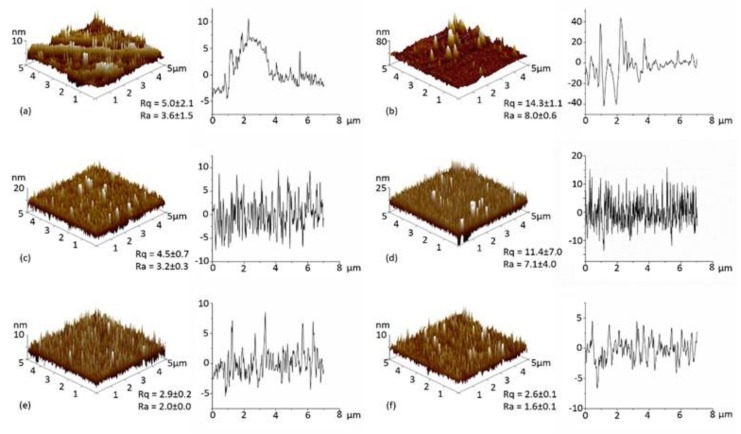
AFM 3D images (5 × 5 µm^2^) and depth profiles of (**a**) PET; (**b**) PETox; (**c**) PET-DET3; (**d**) PET-PTMSPA; (**e**) PET-DET3/NO and (**f**) PET-PTMSPA/NO. Rq and Ra values are given in nm.

The surface topography of the PET, PETox, PET-DET3, PET-DET3/NO, PET-PTMSPA and PET-PTMSPA/NO surfaces was examined by AFM, and representative images with associated roughness values are displayed in Figure 4. Pristine PET has a fairly smooth topography (*R_a_* = 3.2 nm). After plasma treatment, plasma-induced etching of the surface is observed by an increase in the roughness values. Upon silanisation, a decrease in roughness was seen for PET-DET3 in comparison to PETox. A large variation in roughness values was observed for PET-PTMSPA; which is hypothesised to be due to the ease of aminosilanes forming inhomogeneous layers through solution phase deposition [54]. The roughness of both diazeniumdiolate tethered PET surfaces decreased in comparison to silanised surfaces.

#### 3.3.2. SE

**Figure 5:**
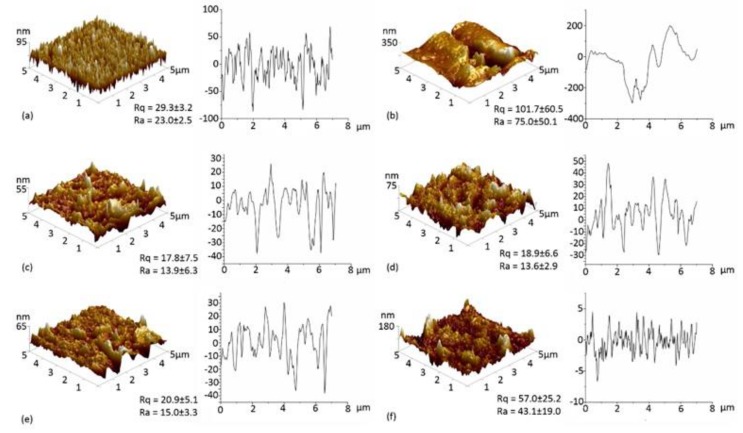
AFM 3D images (5 × 5 µm^2^) and depth profiles of (**a**) SE; (**b**) SEox; (**c**) SE-DET3; (**d**) SE-PTMSPA; (**e**) SE-DET3/NO and (**f**) SE-PTMSPA/NO. *R_q_* and *R_a_* values are given in nm.

The surface topography of the SE, SEox, SE-DET3, SE-DET3/NO, SE-PTMSPA and SE-PTMSPA/NO surfaces was examined by AFM, and representative images with associated roughness values are displayed in Figure 5. Pristine SE has a fairly rough surface (*R_a_* = 23.0 nm). After plasma treatment, large cracks in the polymer can be seen (Figure 5b). This is consistent with the XPS analysis indicating that the SE forms a brittle inorganic silica outer layer, which can form cracks as previously reported in the literature [53,55,56,57,58]. Although silanised and diazeniumdiolate-tethered SE surfaces exhibited no significant difference (*p* < 0.05) in surface roughness, a less uniform array of peaks and troughs can be seen when compared to pristine SE. This is due to agglomerates of silane and resulting diazeniumdiolate found on the surface.

### 3.4. NO Release: Electrochemical Detection

#### 3.4.1. PET

**Figure 6:**
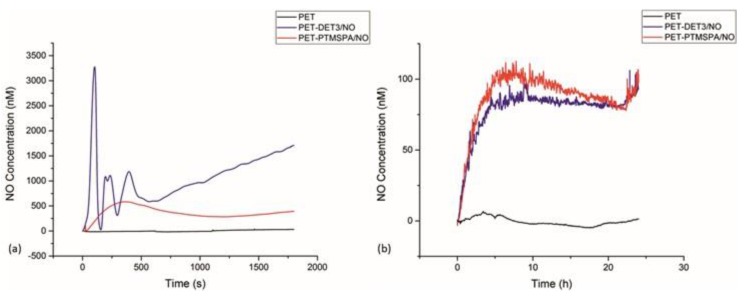
NO release profiles for diazeniumdiolate-tethered PET at (**a**) pH 4 and (**b**) pH 7 determined by electrochemical detection.

Nitric oxide release was monitored for PET-DET3/NO and PET-PTMSPA/NO at ambient temperature (25 °C) in real time via electrochemical detection at pH 4 and 7.4, as shown in Figure 6. Not all pristine substrates and silanised control substrates release NO. At pH 4, a difference was observed for the NO payload, which was dependent on the nature of the silane precursor (DET3 vs. PTMSPA). For PET-DET3/NO, an initial burst release was observed with 3250 nM of NO measured in under 2 min. Two smaller bursts of NO release were observed immediately after, each to a maximum of approximately 1000 nM. A steady, continuous rise was then observed for the remainder of the analysis. PET-PTMSPA/NO released less NO over a longer period of time, gradually rising to a peak of 560 nM after 7 min and then flattening out at approximately 250 nM after 16 min. Lower, steadier rates of release were observed over 24 h at pH 7.

#### 3.4.2. SE

**Figure 7:**
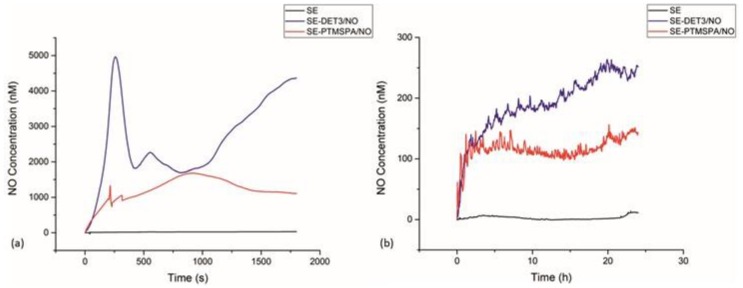
NO release profiles for diazeniumdiolate-tethered SE at (**a**) pH 4 and (**b**) pH 7 determined by electrochemical detection.

Nitric oxide release was monitored for SE-DET3/NO and SE-PTMSPA/NO at ambient temperature (25 °C) in real time via electrochemical detection at pH 4 and 7.4 as shown in Figure 7. Not all pristine substrates and silanised control substrates release NO. Again for SE-DET3/NO, at pH 4, a continuous initial burst release led to a measurement of 5000 nM of NO just after 4 min. In the same manner as PET-DET3/NO, a steady continuous rise of NO was then observed for the remainder of the measurement. For SE-PTMSPA, NO was released steadily, reaching a peak of 1600 nM after 17 min. Lower, steadier rates of release were observed over 24 h at pH 7.

### 3.5. Bacterial Response

**Figure 8:**
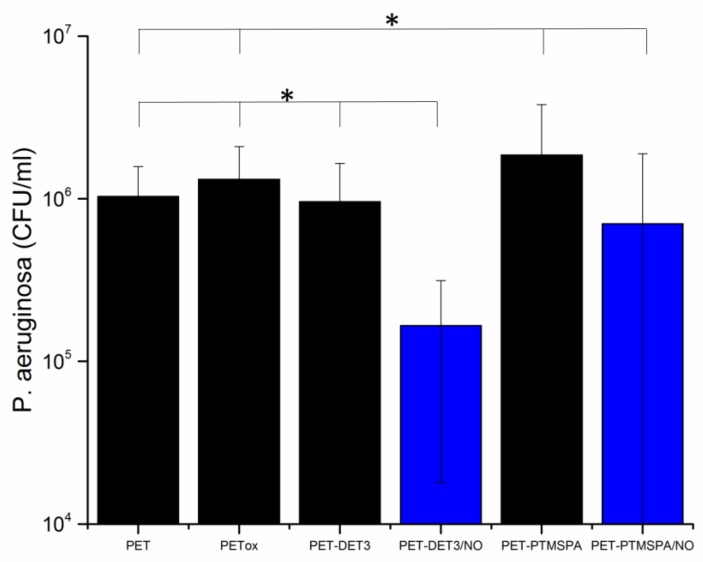
Viable *P. aeruginosa* cell counts (CFU/mL) after 24 h of biofilm growth on PET surfaces. Black bars indicate control surfaces; blue bars indicate NO-releasing surfaces. The symbol * indicates that all NO-releasing surfaces are statistically significantly different from all corresponding control surfaces at *p* < 0.05.

**Figure 9:**
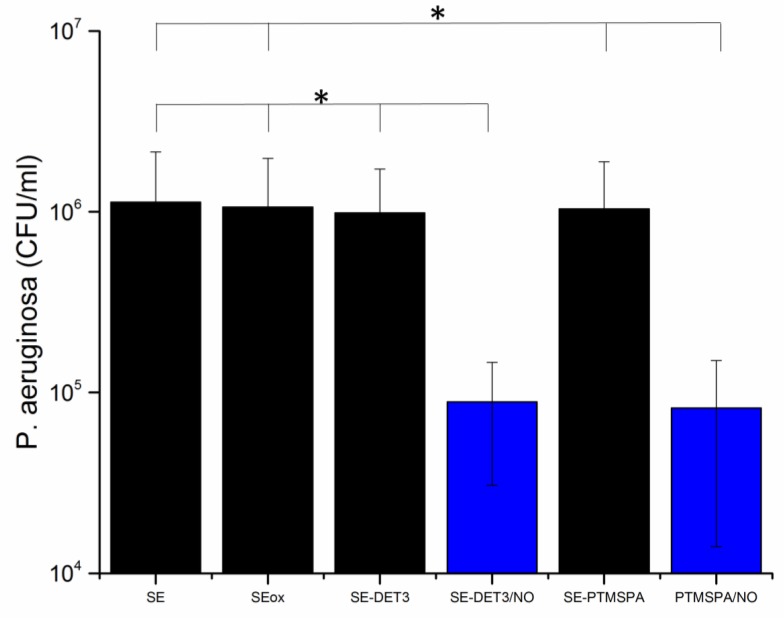
Viable *P. aeruginosa* cell counts (CFU/mL) after 24 h of biofilm growth on SE surfaces. Black bars indicate control surfaces; blue bars indicate NO-releasing surfaces. The symbol * indicates that all NO-releasing surfaces are statistically significantly different from all corresponding control surfaces at *p* < 0.05.

To investigate the antibacterial activity against *P. aeruginosa,* a colony forming unit (CFU) biofilm assay was carried out. The bacteria were incubated with the surfaces for 24 h in LB broth to allow a biofilm to form. Remaining viable bacteria from the surface were then counted to test the efficacy of the NO-releasing surfaces in biofilm prevention. The results are given in Figure 8 and Figure 9 for PET and SE, respectively. For all diazeniumdiolate-tethered polymers, a statistically-significant (*p* < 0.05) reduction in CFU count, compared to pristine, plasma-treated and corresponding silane-tethered control substrates demonstrates that all NO-releasing polymers are capable of disrupting *P. aeruginosa* biofilms. Specifically, in the case of PET-DET3/NO and PET-PTMSPA/NO, 83% and 62% reduction in viable bacteria was observed. Similarly, for the SE surfaces, 92% reduction was observed for both SE-DET3/NO and SE-PTMSPA/NO. 

## 4. Discussion

NO is an endogenously produced molecule that plays an important role in the host antimicrobial response [25]. Once bacterial infection occurs, cytokines signal macrophages to produce NO, which acts as a potent oxidising agent causing oxidative stress via a plethora of reactive nitrogen intermediates. For example, NO reacts with superoxide (also produced by macrophages) to produce peroxynitrite (^−^OONO), which can damage the cell membrane due to lipid peroxidation [59]. The reactive nitrogen species formed by NO are also able to damage DNA and denature proteins [60]. The antimicrobial effects of NO in solution-based assays was first observed by Raulli and co-workers who showed low molecular weight diethylenetriamine-derived diazeniumdiolate (DETA/NO) to have a bactericidal efficacy against a range of Gram-positive and Gram-negative species [61]. Schoenfisch and Meyerhoff doped LMW diazeniumdiolates into hydrophobic polymers [62,63]. However, there were concerns of leaching of by-products and their potential toxicity. 

To circumvent the issue of leaching and toxic metabolites, several research groups have covalently tethered diazeniumdiolates into NO-releasing coatings. Schoenfisch’s group has developed a series of sol-gel and xerogel coatings that are loaded with diazeniumdiolates [40,41,42,64,65]. These covalently-tethered diazeniumdiolate coatings were found to be effective in decreasing bacterial adhesion of *S. aureus*, *Escherichia coli* and *P. aeruginosa*. The studies showed that the maximal flux of NO released from the coatings occurred shortly after immersion in buffer followed by a gradual release over time. The duration and amount of release was based on the quantity and type of aminosilane used in the xerogel/sol-gel coating. For instance, the 40% *N*-(6-aminohexyl)aminopropyltrimethoxysilane/Isobutyltrimethoxysilanecoatings released detectable quantities of NO up to 20 days [42]. While promising, increasing the aminosilane concentration to increase the NO release was limited by the xerogel stability [42]. 

The experimental approach taken in this study was to determine whether a simple aminosilane coating on polymer surfaces could be tethered with diazeniumdiolates and whether the release could be controlled in terms of the type of aminosilane used. For our surfaces, we found that the type of aminosilane used and indeed the substrate had an effect on the flux and payload of NO released. The DET3 silane has a triamine precursor (diethylenetriamine) with two secondary amines that can be used to tether diazeniumdiolates. The PTMSPA has only one secondary amine that can be used to tether the diazeniumdiolate. Indeed, the NO release from the PET-DET3/NO and SE-DET3/NO was higher than PET-PTMSPA/NO and SE-PTMSPA/NO at pH 4 as seen in Figure 6 and Figure 7. From Figure 7a, it can also be seen that at pH 4, SE-DET3/NO had an initial burst release of 5000 nM compared with a lower initial release concentration by PET-DET3/NO (Figure 6a). This is attributed to SE being porous, and as a result, it is hypothesised that some of the aminosilane precursor is doped within the subsurface of the polymer. As the diazeniumdiolate reaction occurs under high pressure, NO is able to reach the doped aminosilane precursor and form a diazeniumdiolate. This would account for the significant increase in the concentration of NO on SE vs. PET. The shelf-life or stability of the NO-releasing coating was analysed over a 14-day period after storage in air and at −20 °C. The payload of NO release was significantly reduced when the substrates were left in air, indicating limited stability under atmospheric conditions. Substrates left in the freezer showed a slight decrease in payload up to 14 days, indicating that the samples may be stored cold. Additional experiments exploring ways of increasing the stability of these coatings is the focus of another study. 

Diazeniumdiolate dissociation to NO is the reverse of its formation as shown in Figure 1 [66]. Therefore, the decomposition is based on the initial protonation of the amine functionality of the diazeniumdiolate, which yields up to 2 mol of NO. As such, the decomposition of the diazeniumdiolate moiety is dependent on the pKa of the secondary amine that is used to form the diazeniumdiolate, and the decomposition reaction is accelerated when the molecule comes into contact with water or another proton source (protonation of the amino nitrogen) [66], an increase in temperature [67] or a shift in the equilibrium towards the aminosilane precursor vs. the diazeniumdiolate (Figure 1) [66]. The pKa of diethylenetriamine (the precursor used to form the DET3/NO) is 10.45, while the pKa of aniline (the precursor of PTMSPA/NO) is 4.6. Based on the approximate pKa values, DET3 is more easily protonated and will decompose faster at pH 4 than PTMSPA. This is evident from the NO releasing profiles of PET-DET3/NO and SE-PTMSPA/NO at pH 4 (Figure 6a and Figure 7a), which show DET3/NO surfaces to have a faster burst release followed by slower release than the PTMSPA/NO surfaces. Furthermore, Nablo et al. have shown that DET3/NO xerogels exhibited an enhanced diazeniumdiolate conversion efficiency due to the improved deprotonation resulting from the additional amines [64]. Nablo et al. have also shown that a hydrophobic substrate such as PVC can hinder the diffusion of water, which affects the decomposition rate, by reducing the initial NO flux and prolonging the release duration of NO [41].

Although studying microbes in planktonic, nutrient-rich batch cultures is useful for antimicrobial screening, in vivo infections are typically caused by bacterial biofilms [45,46]. In this study, more persistent cultures of *P. aeruginosa* (PA14) grown for a 24-h incubation period have been utilised. This assay more closely represents biofilm formation than the more commonly-used 30-min assay, which only represents the very early stages of bacterial attachment. All four diazeniumdiolated surfaces, PET-DET3/NO, PET-PTMSPA/NO, SE-DET3/NO and SE-PTMSPA/NO, reduced bacterial colonisation and biofilm formation over 24 h. The reduced bacterial load after growth for the SE surfaces was probably due to the higher initial rate of NO release, which may be crucial for fighting the early stages of bacterial colonisation and biofilm formation. It is envisaged that a prolonged and extended NO release in the slower phase can be effective for avoiding the recovery of bacterial growth, and this is the focus of subsequent studies.

## 5. Conclusions

This paper reports on the antibacterial nature of nitric oxide, which herein has shown to actively prevent *P. aeruginosa* biofilm formation when administered through different NO-releasing polymers on PET and SE. The NO payload and release were controlled by the aminosilane precursor, as DET3 has two secondary amine sites and PTMSPA only one. All NO-releasing coatings in this study were shown to significantly reduce *P. aeruginosa* adhesion over 24 h with the efficacy being a function of the aminosilane modification and the underlying substrate. These NO-releasing polymers demonstrate the potential and utility of this facile coating technique for preventing biofilms for indwelling medical devices. Future work will report on broadening the utility of these coatings in order to lengthen and optimise release under physiological conditions.

## Figures and Tables

**Table 1:** Static water contact angle measurements of silanised and diazeniumdiolate-tethered PET and SE.

Surface	Contact Angle (°)	
	PET	SE
**Pristine**	88.1 ± 1.0	113.0 ± 2.3
**Plasma treated**	19.7 ± 1.8	11.0 ± 1.2
**DET3**	90.2 ± 0.9	116.6 ± 1.8
**DET3/NO**	79.0 ± 0.4	108.8 ± 2.0
**PTMSPA**	88.5 ± 0.3	119.2 ± 1.5
**PTMSPA/NO**	81.7 ± 1.9	108.6 ± 2.8

**Table 2:** XPS derived at% of C 1s, O 1s, N 1s and Si 2p regions for PET and SE surfaces.

Sample	at %			
	C 1s	O 1s	N 1s	Si 2p
**PET**	73.0 ± 0.4	27.0 ± 0.4	-	-
**PETox**	66.0 ± 0.2	34.0 ± 0.2	-	-
**PET-DET3**	56.0 ± 1.1	22.5 ± 0.2	4.7 ± 0.3	16.8 ± 1.2
**PET-DET3/NO**	57.2 ± 0.3	26.9 ± 0.1	5.7 ± 0.7	10.2 ± 0.4
**PET-PTMSPA**	61.7 ± 1.1	25.4 ± 0.2	3.3 ± 0.3	9.6 ± 0.6
**PET-PTMSPA/NO**	59.6 ± 0.6	25.5 ± 0.6	4.5 ± 0.9	10.4 ± 1.4
**SE**	38.2 ± 1.5	32.2 ± 1.2	-	29.6 ± 0.3
**SEox**	35.4 ± 1.5	35.1 ± 1.6	-	29.6 ± 0.5
**SE-DET3**	41.3 ± 1.2	28.4 ± 1.0	3.7 ± 0.5	26.6 ± 0.7
**SE-DET3/NO**	33.8 ± 0.9	35.1 ± 0.7	2.4 ± 0.2	29.4 ± 0.7
**SE-PTMSPA**	40.5 ± 1.5	30.0 ± 1.3	1.8 ± 0.0	27.7 ± 0.3
**SE-PTMSPA/NO**	37.1 ± 0.4	33.4 ± 0.4	1.6 ± 0.1	28.0 ± 0.0

**Table 3:** XPS-derived curved-fitted C 1s and N 1s components for PET surfaces.

Sample	at %					
	C 1s			N 1s		
	C–H, C–C	O–CH_2_CH_2_	O=C–O	N–H	N+	N–O
**PET**	59.6 ± 0.2	24.0 ± 0.2	16.5 ± 0.2	-	-	-
**PETox**	41.1 ± 0.6	37.4 ± 0.6	21.5 ± 0.1	-	-	-
**PET-DET3**	54.8 ± 3.7	40.4 ± 3.4	4.8 ± 0.7	52.0 ± 0.9	48.0 ± 0.9	-
**PET-DET3/NO**	62.0 ± 2.7	26.4 ± 2.1	11.6 ± 0.6	34.7 ± 0.5	32.7 ± 0.2	32.7 ± 0.3
**PET-PTMSPA**	69.9 ± 0.3	19.4 ± 0.8	10.7 ± 0.7	68.7 ± 1.2	31.3 ± 1.2	-
**PET-PTMSPA/NO**	66.2 ± 1.7	25.4 ± 0.9	8.6 ± 0.9	28.1 ± 3.1	35.9 ± 1.5	35.9 ± 1.5

**Table 4:** XPS-derived curved-fitted C 1s and N 1s components for SE surfaces.

Sample	at %				
	C 1s		N 1s		
	C–H, C–C, C–Si	C–O	N–H	N+	N–O
**SE**	100.0 ± 0.0	-	-	-	-
**SEox**	82.2 ± 2.9	17.8 ± 2.9	-	-	-
**SE-DET3**	62.0 ± 6.2	38.0 ± 6.2	54.8 ± 3.2	45.2 ± 3.2	-
**SE-DET3/NO**	77.3 ± 5.3	22.7 ± 5.3	39.3 ± 0.2	30.4 ± 0.1	30.4 ± 0.1
**SE-PTMSPA**	70.9 ± 2.6	29.1 ± 2.6	66.6 ± 0.2	33.4 ± 0.2	-
**SE-PTMSPA/NO**	79.0 ± 2.5	21.0 ± 2.5	43.8 ± 4.5	28.1 ± 2.2	28.1 ± 2.2
